# Detection is unaffected by the deployment of focal attention

**DOI:** 10.3389/fpsyg.2013.00284

**Published:** 2013-05-27

**Authors:** Jeff Moher, Brandon K. Ashinoff, Howard E. Egeth

**Affiliations:** Department of Psychological and Brain Sciences, Johns Hopkins UniversityBaltimore, MD, USA

**Keywords:** focal attention, perception, salience, locus of selection, priming

## Abstract

There has been much debate regarding how much information humans can extract from their environment without the use of limited attentional resources. In a recent study, Theeuwes et al. (2008) argued that even detection of simple feature targets is not possible without selection by focal attention. Supporting this claim, they found response time (RT) benefits in a simple feature (color) detection task when a target letter's identity was repeated on consecutive trials, suggesting that the letter was selected by focal attention and identified prior to detection. This intertrial repetition benefit remained even when observers were required to simultaneously identify a central digit. However, we found that intertrial repetition benefits disappeared when a simple color target was presented among a heterogeneously (rather than homogeneously) colored set of distractors, thus reducing its bottom–up salience. Still, detection performance remained high. Thus, detection performance was unaffected by whether a letter was focally attended and identified prior to detection or not. Intertrial identity repetition benefits also disappeared when observers were required to perform a simultaneous, attention-demanding central task (Experiment 2), or when unfamiliar Chinese characters were used (Experiment 3). Together, these results suggest that while shifts of focal attention can be affected by target salience, by the availability of excess cognitive resources, and by target familiarity, detection performance itself is unaffected by these manipulations and is thus unaffected by the deployment of focal attention.

Humans often need to extract information from a complex visual world in order to accomplish behavioral goals. Attentional mechanisms provide one solution to this problem, allowing observers to select a subset of information from their surrounding environment for more detailed processing. However, in some cases information may be accessible without the use of limited attentional resources. In the present paper, we will consider whether simple feature targets can be detected without the deployment of focal attention.

There is a long-standing debate regarding the locus of the selection process. Early selection proponents (Broadbent, 1958; Treisman, 1964; Neisser, 1967) argue that only a very limited amount of information is available prior to selection. Late selection proponents (Cherry, 1953; Deutsch and Deutsch, 1963; Allport, 1977) argue that more detailed processing, such as semantic encoding, may occur in the absence of attention. More recent models suggest that the locus of selection may be flexible; for example, the demands of the task may determine the locus of selection (e.g., Yantis and Johnston, 1990). The perceptual load of the display may also affect the locus of selection (e.g., Lavie et al., 2004), although an alternative “dilution” account may explain these perceptual load effects (e.g., Tsal and Benoni, 2010).

Even strict early selection models of attention allow for some “preattentive” processing (e.g., Treisman and Gelade, 1980) in which some basic, low-level information is processed without the use of limited attentional resources. This preattentively acquired information is then available for use during subsequent cognitive processes. For example, when observers search for a target defined by one or more known properties, such as color, information from preattentive processing may be used to guide that selection process (e.g., Treisman and Sato, 1990; Wolfe, 1994; see Wolfe, 2007 for a more detailed description of guidance).

While most models of attention agree that some information is encoded preattentively, there is less consensus regarding whether observers have direct access to that information. According to Treisman and Gelade's (1980) Feature Integration Theory (FIT), individual “feature maps” register the presence of individual low-level features (e.g., color) rapidly and efficiently throughout the entire visual field. Observers can directly access these feature maps to detect a signal indicating the presence of a given feature. This direct access allows for detection of simple feature targets without needing to select those targets with a focal shift of spatial attention. Some data have supported this theory by demonstrating that detection of singleton targets (e.g., Braun and Sagi, 1990; Braun and Julesz, 1998; but see Joseph et al., 1997) or simple feature targets (e.g., Luck and Ford, 1998) is unaffected when a secondary, attention-demanding task is added concurrently with a primary task.

The Guided Search model of attention (Wolfe, 1994) is a proposed alternative to FIT. According to this model, feature maps are combined into an activation map, and attention is directed to the location with the greatest signal in that activation map. Therefore, even in an efficient search, focal attention must be directed to the location of the target prior to detection of that target by the observer. There is empirical support for the Guided Search model as well (e.g., Joseph et al., 1997; Kim and Cave, 1995). For example, Joseph et al. (1997) found that performance on a singleton detection task suffered when it was presented during an “attentional blink” (AB) period. The AB is a period of time after one target is presented in a central stream when attentional resources are diverted, thus making processing of a second target more difficult (e.g., Chun and Potter, 1995). Joseph et al.'s result thus suggests that attentional resources are necessary even for a simple pop-out detection task (but see Egeth et al., 2008, for conflicting results). However, an alternative account of the AB suggests it may reflect active suppression of incoming visual input rather than an absence of attentional resources (Olivers and Meeter, 2008), meaning that Joseph et al.'s results may be attributable to an active suppression process.

## A new method

In a recent study by Theeuwes et al. (2008), participants were asked to report whether a single red letter was present among a ring of otherwise gray letters. Participants responded more quickly when the identity of the target letter was repeated than when it was not. This is consistent with previous studies demonstrating that repetition of the identity of an attended stimulus can speed processing of that stimulus, even when identity is not task-relevant (e.g., Huang et al., 2004). Thus, participants must have processed the identity of the target letter at some level prior to executing a response indicating its presence. This effect persisted even when participants were required to identify a centrally presented digit, a task intended to tax attentional resources.

Critically, repetition of the identity of non-target letters had no effect on responses (for example, if a non-target gray “W” became the red target “W” on the next trial). This suggested that the identity of each and every individual letter was not available preattentively. The implication is that participants processed the identity of the target letter by selecting that letter with focal attention. Therefore, intertrial identity repetition benefits could only occur if the target letter was selected by focal attention, and thus identified, prior to the detection response. The authors concluded that this shift of focal attention was a necessary precondition for detection of the target letter.

## The present study

Theeuwes et al. (2008) assumed that because a shift of attention occurred prior to the detection response, attention is a necessary precursor of detection. However, another possibility is that shifts of focal attention may have occurred but they may be unrelated to detection. That is, whether and when a shift of focal attention occurs may not affect detection performance, suggesting that detection relies on an independent mechanism. In the present study, we examine this possibility by applying the method of Theeuwes et al. (2008) to various simple feature target detection tasks in which deployment of focal attention is affected by manipulation of stimulus properties and task demands. We assess deployment of focal attention via intertrial repetition effects, and determine whether detection performance is affected by whether intertrial repetition benefits occur.

## Experiment 1

In Theeuwes et al. (2008), the target, if present, was a color singleton. The authors concluded that focal attention is a necessary precursor of detection of simple feature targets. However, bottom–up salient items, such as color singletons, have been shown in some cases to capture attention regardless of an observer's intentions (e.g., Theeuwes, 1991; Bacon and Egeth, 1994, Experiment 1). Therefore, the deployment of focal attention may have occurred because of the target's bottom–up salience, and not because attention is required for detection.

In Experiment 1, we varied the bottom–up salience of the target by manipulating the color heterogeneity of the display; while singleton targets presented among a homogeneous set of distractors provide a strong bottom–up signal that can bias attention, no item provides a strong bottom–up signal (or “pops out”) in a heterogeneously colored display (e.g., Duncan and Humphreys, 1989; Wolfe, 1992, 1994). We conducted a separate pilot study which confirmed that search times for the target were efficient even when the target was not a color singleton^1^, as would be expected in a simple feature search task (e.g., Duncan, 1989; Duncan and Humphreys, 1989; Wolfe, 1994). However, in the non-singleton-target condition the bottom–up salience of the target was considerably reduced, and thus the target was unlikely to capture attention in a purely bottom–up manner. This design allowed us to explore the possibility that the repetition effects in Theeuwes et al.'s study occurred because the target automatically captured attention due to its bottom–up salience, and not because focal attention necessarily precedes detection.

### Methods

#### Participants

Thirty-two Johns Hopkins University undergraduate students (mean age = 19.8 years; 18 male) with normal or corrected-to-normal visual acuity and normal color vision participated for course credit in sessions lasting 30 min. Participants gave informed consent, and the protocol was approved by the Johns Hopkins Homewood Institutional Review Board.

#### Apparatus

Stimulus presentation and data analysis were performed using programs written in Matlab (Mathworks) and using PsychToolbox software (Brainard, 1997).

#### Stimuli

On each trial, 8 English letters appeared, arranged in a circle surrounding the center of the display. At a viewing distance of 42 cm, each letter subtended a visual angle of 1°, and the radius of the circle that the letters formed subtended 6.35° of visual angle.

**Figure 1:**
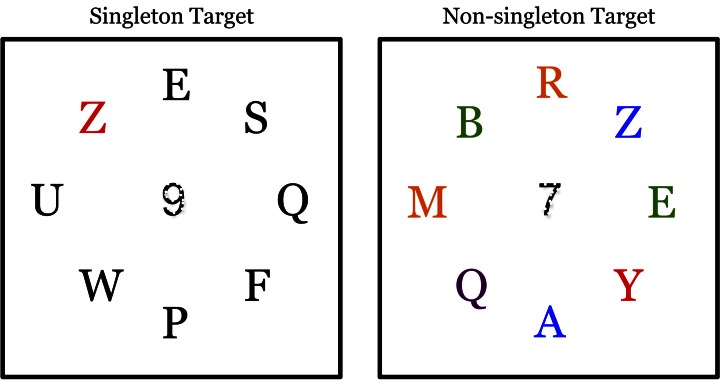
**In both conditions, participants indicated the presence or absence of a red letter.** In the singleton-target condition, all remaining letters were colored white on a black background (black and white are reversed in the figures for clarity). In the non-singleton-target condition, the remaining letters were heterogeneously colored. The display was on for 120 ms.

Following Theeuwes et al. (2008), participants had to indicate with a key press whether or not a red target letter was present. Each trial was randomly assigned as either target present or target absent. In the singleton-target condition, all seven non-target letters were colored white. In the non-singleton-target condition, non-target letters were heterogeneously colored, consisting of a combination of white, green, blue, pink, and yellow letters (see Figure 1). A white digit (1–8) obscured by dots (as in Theeuwes et al., 2008) appeared on each trial in all conditions at fixation, subtending 1° of visual angle. However, participants were instructed to ignore the central number (it was present as a perceptual control for an alternate set of experiments not reported in the present paper).

#### Design and procedure

Participants were randomly assigned to either the singleton-target or non-singleton-target condition for the duration of the experiment. Each trial began with a display of a white fixation cross at the center of the screen subtending 1° of visual angle for 1 s. Following this, the primary stimulus display featuring the digit at the center and 8 English letters in a circular array surrounding the center appeared for 120 ms. Participants subsequently indicated whether or not a red letter had been present with a keypress (“z” for present, “x” for absent). There was a 500 ms intertrial interval during which a blank black screen was presented.

When a red target letter was present on consecutive trials (roughly 25% of all trials), there was a 50% chance the target letter identity was repeated. Therefore, ~12.5% of all trials included a repeated target letter. There were 10 blocks of 64 trials each in the experiment, the first of which was a practice block.

### Results and discussion

We eliminated all responses faster than 100 ms and subsequently used a modified recursive trimming procedure (Van Selst and Jolicoeur, 1994) to remove outliers in each experimental condition. This resulted in an elimination of 1.2% of all trials. All error trials were removed for response time (RT) analysis (3% of all trials). Because we were comparing intertrial effects resulting from repeated target properties, the following analyses only include trials where a target was present on consecutive trials (i.e., trials *N-1* and *N*) and the observers' response on the previous trial (i.e., on trial *N-1*) was correct.

We conducted a 2 × 2 mixed-design ANOVA with a between-subjects factor of target type (singleton vs. non-singleton) and a within-subjects factor of target identity repetition (identity repeated vs. not repeated) for measures of error rate (ER) and RT. Performance accuracy was high overall (97%).

There was no main effect of target type on RT or ER (*p*s > 0.1). Thus, the bottom–up salience of the target did not impact overall detection performance. There were no main effects or interactions for ER on any analyses; thus, the rest of the section focuses on RT measures only (ERs for this and subsequent experiments are reported in Table 1).

**Table 1:** **Error rates across all experiments**.

**Experiment**	**Identity repeated**	**Identity not repeated**	**Overall performance**
Extaperiment 1			
Singleton condition	3.55	3.53	2.62
Non-singleton condition	2.42	3.37	3.32
Experiment 2	3.1	3.59	4.13
Experiment 3A	1.25	2.27	2.12
Experiment 3B			
English letters	2.15	4.21	3.44
Chinese letters	1.11	2.97	2.9

Percentage error rates for all experimental conditions in all three experiments (first two columns), and overall error rates for all three experiments (third column). Overall error rates calculated across all trials in a given experiment (or language type, in the case of Experiment 3B) regardless of whether target presence, identity, or language type was repeated across trials. Because this includes many trials that were not included in calculations of identity repeated and identity not repeated trials, this number does not necessarily equal the mean of the error rates in those two conditions.

**Figure 2:**
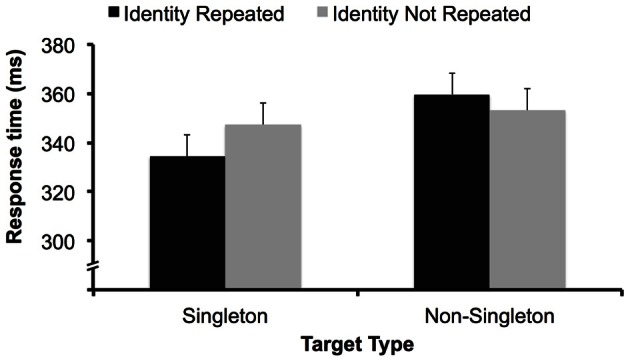
**Data from Experiment 1.** Participants responded more quickly when the target letter identity was repeated in the singleton-target condition. There was no effect of target identity repetition in the non-singleton-target condition. Error bars represent 95% confidence intervals for the singleton-target condition and non-sinlgeton-target conditions separately. (e.g., Loftus and Masson, 1994).

If focal attention necessarily precedes detection in a fixed manner, letter identification likely also occurs prior to detection (as in Theeuwes et al., 2008), and we would expect a main effect of target identity repetition. However, there was no main effect of target identity repetition, *F*_(1, 30)_ < 1. There was, however, an interaction between target type and target identity repetition, *F*_(1, 30)_ = 5.68, *p* < 0.05 (Figure 2). We conducted simple main effects analyses to interpret this interaction.

In the singleton-target condition, responses were significantly faster when the target was repeated (334 ms) than when the target identity was not repeated (347 ms), *F*_(1, 15)_ = 5, *p* < 0.05, replicating (Theeuwes et al., 2008) and suggesting that the target letter was selected by focal attention prior to the execution of a detection response in this condition. In a separate analysis, we found there was no effect of a non-target letter on the previous trial becoming the target on the current trial (also replicating Theeuwes et al., 2008), *F*_(1, 15)_ < 1. This demonstrates that participants were not identifying all the letters on the screen preattentively, but instead only processing the identity of the target letter after selecting that letter with focal attention.

In the non-singleton-target condition, there was no benefit to repeating target identity; instead, responses were numerically *slower* in trials where target identity was repeated (360 ms) vs. when it was not (353 ms), though this 7 ms difference did not approach significance, *F*_(1, 15)_ = 1.28, *p* > 0.1. Thus, when the target was not a color singleton, the identity of the target letter was *not* fully processed prior to execution of a detection response.

From these results, we can conclude that the selection process itself must have differed between the two conditions even though detection performance did not (no overall difference in RT or ER). Although a null result cannot be taken as definitive evidence, the data nonetheless suggest that the target letter was not selected by focal attention prior to detection in the non-singleton-target condition. We discuss more detailed theoretical accounts of these data in the discussion; however, what these data unambiguously show is that the process of detecting a simple feature target is not affected by changes in the deployment of focal attention. Therefore, it may be the case that focal attention is not a necessary precondition for simple feature target detection, and Theeuwes et al.'s results may instead be explained by the target's bottom–up salience.

## Experiment 2

In Luck and Ford (1998), as well as in Theeuwes et al. (2008), participants had to identify a central digit obscured by dots while also performing a simple detection task. By adding a difficult second task at the center of the screen, the authors of those studies reasoned that participants would no longer have excess attentional resources available to devote to the simple feature detection task. Luck and Ford (1998) found that the N2PC disappeared when the second central task was added, leading them to conclude that focal attention was not necessary for the feature detection task, as it was in evidence only when there were no other demands on attention. Theeuwes et al. (2008), on the other hand, found that adding a central task had no effect on intertrial facilitation when the identity of the target character was repeated, suggesting that focal attention was still directed to the peripheral target even when attentional resources were being taxed by the central task.

However, it is possible that a number obscured by dots was not the ideal secondary task to sufficiently tax attentional resources. The presence of obscuring dots imposes a data limitation on processing (cf. Norman and Bobrow, 1975), meaning that additional attentional resources might not overcome the lack of sensory information coming from the letter. In data-limited tasks like this, participants may not devote additional attentional resources to the digit if they are having trouble identifying it because doing so will not improve their performance.

In Experiment 2, we introduced a “resource-limited” central task instead in order to more effectively tax attentional resources. In resource-limited tasks, task difficulty *can* be overcome with the use of additional attentional resources (cf. Norman and Bobrow, 1975). Thus, inclusion of a resource-limited task would increase the load on available cognitive resources, potentially eliminating an unnecessary shift of attention in the detection task. We had participants search for a rotated “T” among rotated “L”s near the center of the screen while simultaneously determining whether a simple feature target was present. This type of spatial-configuration task was shown to be attentionally demanding by Huang and Pashler (2005), and thus could be described as a “resource-limited” task.

### Methods

Thirty-two Johns Hopkins University undergraduate students (mean age = 19.3 years; 13 male) with normal or corrected-to-normal visual acuity and normal color vision participated in sessions for course credit lasting 30–60 min. Participants gave informed consent, and the protocol was approved by the Johns Hopkins Homewood Institutional Review Board.

**Figure 3:**
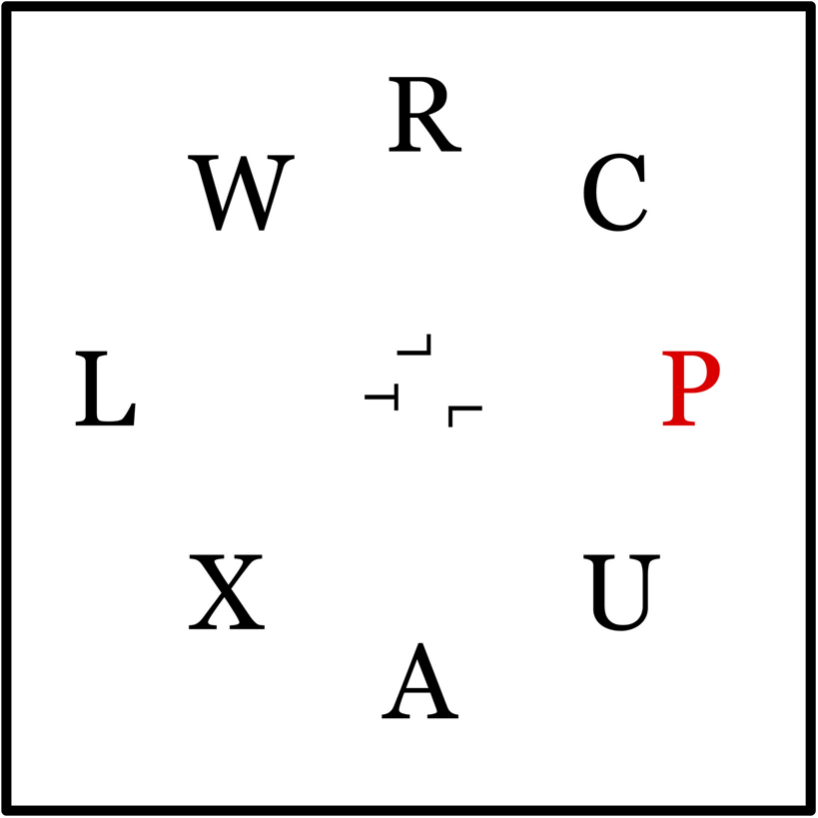
**After indicating the presence or absence of a red letter, participants were required to report the orientation of a rotated “T” presented among rotated “L”s in the center of the display (the “T” was either facing left or facing right).** The second response was untimed. The display was on for 120 ms.

The peripheral task was identical to the singleton-target condition in Experiment 1. In addition, at a random location inside an imaginary circle with a radius of 3.22° of visual angle surrounding the center of the screen, there was a single rotated “T” subtending 0.28° of visual angle. There were between 1 and 4 rotated “L”s present in the circle as well. The number of “L”s was kept consistent throughout a block, and there were two blocks each of the four possible numbers (1, 2, 3, or 4), of distractor “L”s resulting in 8 total blocks of trials, preceded by one practice block with two distractor “L”s. The order of blocks was assigned randomly across participants. Each “L” was the same size as the “T,” taking up 0.28° of visual angle. All items were displayed on the screen for 120 ms (see Figure 3).

Participants first responded to the presence or absence of red by pressing either the “z” key or the “x” key with their left hand. Following that, participants were told to indicate which direction the “T” was pointing, either to the right or to the left. Using the number pad with their right hand, participants pressed the “4” key if the “T” was pointing left, and the “6” key if it was pointing right. Participants were encouraged to make a speeded response to the presence or absence of red, but they were told that their response to the orientation of the “T” was an untimed response. There were 9 blocks of 64 trials each in the experiment, the first of which was a practice block.

### Results and discussion

We eliminated all responses faster than 100 ms and subsequently used a modified recursive trimming procedure (Van Selst and Jolicoeur, 1994) to remove outliers in each experimental condition. This resulted in an elimination of 0.6% of all trials. Because we were comparing intertrial effects resulting from repeated target properties, the following analyses only include trials where a target was present on consecutive trials, the observers' response to the peripheral task was correct on the previous trial was correct, and the observers' response to the central task on both the previous and current trial was correct. Additionally, all error trials from the peripheral task (4.1% of all trials) were removed from RT analyses. Performance on the central task was 83.7% overall. This was comparable to previous studies using a central task to tax attentional resources (Luck and Ford, 1998; Theeuwes et al., 2008).

Participants still performed very well on the primary task (the detection of the red target), answering correctly on 95.9% of all trials. In a one-way ANOVA comparing accuracy across the two conditions of Experiment 1 and the present experiment as a third condition, there was no main effect of condition on accuracy, *F*_(2, 61)_ = 1.55, *p* > 0.1. Thus, performance accuracy on the detection task was not statistically worse when a secondary, resource-demanding central task was added to the display.

**Figure 4:**
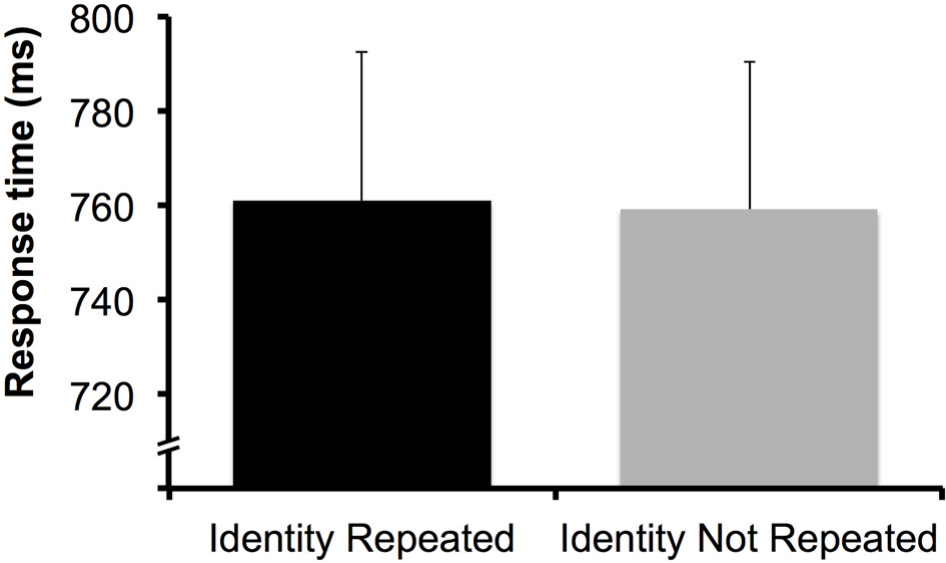
**Data from Experiment 2.** There was no effect of target identity repetition. Error bars represent 95% confidence interval (e.g., Loftus and Masson, 1994).

We conducted a one-way ANOVA with a within-subject factor of target identity repetition (identity repeated vs. not repeated) for measures of ER and RT. RT was practically identical whether or not the target identity was repeated (761 ms for repeated, 759 for non-repeated), *F*_(1, 31)_ < 1 (Figure 4), and there was no effect of repeating target identity on ER (3.1% for repeated, 3.3% for non-repeated), *F*_(1, 31)_ < 1. Unlike in Theeuwes et al. (2008), the addition of a central task eliminated intertrial facilitation effects. This difference in outcomes is likely attributable to the use of a resource-limited task in the present experiment, which depleted available attentional resources more successfully than the number obscured by dots used by Luck and Ford (1998) and Theeuwes et al. (2008).

One possible concern is that the target's identity was processed prior to detection, but that target repetition benefits dissipated because RTs were long overall compared to the first experiment. Theeuwes et al. (2008) found repetition priming effects in a dual-task experiment with long RTs, but the RTs in that study were around 160 ms shorter on average than the RTs in the present experiment (~600 vs. ~760 ms). Other studies, however, have found target repetition benefits for RTs longer than those observed in the present study (e.g., Fecteau, 2007 found priming effects for RTs ~900 ms), and priming effects can persist over periods of time up to ~30 s or longer (e.g., Maljkovic and Nakayama, 1994). Thus, it is reasonable to expect that target repetition benefits might be observed for RTs as long as those found in the present study.

**Figure 5:**
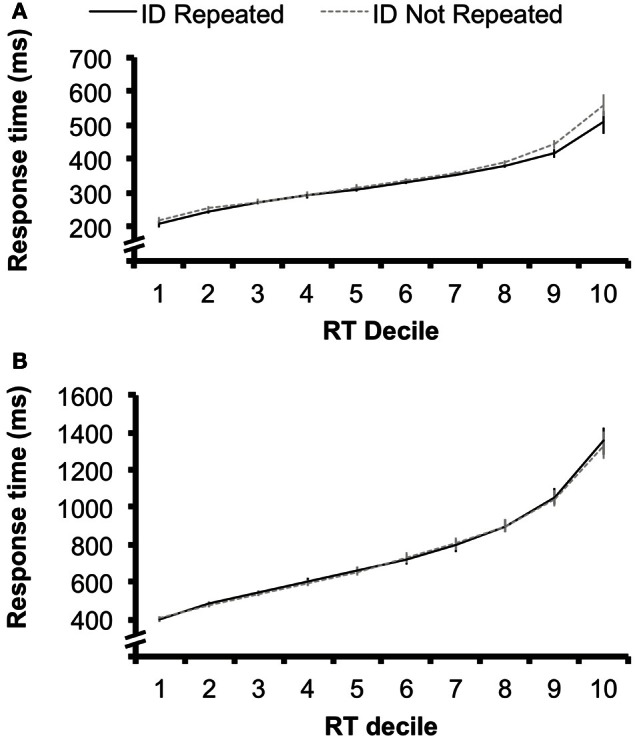
**(A)** Data from the singleton-target condition of Experiment 1 sorted into response time deciles for the target identity repeated (ID Repeated) and target identity not repeated (ID Not Repeated), with the fastest 10% of responses in each condition in Decile 1, and the slowest 10% of responses in Decile 10. The magnitude of the priming benefit did not diminish in slower responses; instead, target identity repetition benefits were larger in magnitude in later deciles in which response times were slower. Error bars represent 95% confidence interval calculated separately for each decile (e.g., Loftus and Masson, 1994). **(B)** The same analysis applied to the data from Experiment 2. Target identity repetition benefits are not observed at any decile. Error bars represent 95% confidence interval calculated separately for each decile (e.g., Loftus and Masson, 1994).

Nevertheless, we re-analyzed the results of the singleton target condition from Experiment 1 by separating the responses from each subject into RT deciles for each condition. This allowed us to determine whether target repetition effects diminished at longer RTs (Figure 5A). We conducted a 2 × 10 ANOVA with factors of target identity repetition (identity repeated vs. not repeated) and decile (1–10) on these RT data. There was an interaction between these two factors, *F*_(9, 135)_ = 3.96, *p* < 0.001, suggesting that the effect of target identity repetition did differ across decile. In Figure 5A, it appears that target repetition benefits do not diminish at longer RTs. If anything, target repetition benefits are larger in magnitude for longer RTs; for example, at deciles 8–10, the average target repetition benefit is 28 ms, while the magnitude of target repetition benefits at deciles 1–7 is 5 ms. A *post-hoc* contrast-contrast interaction analysis revealed that this comparison between the target repetition effect at deciles 1–7 vs. 8–10 was significant, *F*_(9, 135)_ = 20.24, *p* < 0.001. This analysis confirms that target repetition benefits were not diminished at longer RTs, but instead were more pronounced.

To further ensure that target letter identity effects were not obscured by longer RTs, we conducted the same decile analysis for the data from Experiment 2 (Figure 5B). We found no interaction between target identity repetition and decile, *F*_(9, 135)_ < 1, suggesting that even when RTs were comparable in length to those of Experiment 1 (mean RT in decile 1 = 400 ms), there was no effect of target identity repetition.

As with Experiment 1, we cannot definitively conclude from this null effect that attention was not shifted. Nevertheless, these data demonstrate that the inclusion of a resource-limited central task eliminated intertrial identity repetition effects, while detection performance remained high, providing converging evidence that detection performance is unaffected by shifts of focal attention.

## Experiment 3A

Teichner and Krebs (1974) demonstrated that familiar characters may undergo “compulsive encoding,” meaning that they are automatically processed regardless of the task goals of the observer. It could therefore be the case that the use of familiar characters in Theeuwes et al. (2008) biased observers to compulsively encode the target letter, which required a shift of focal attention. In Experiment 3A, we replaced all familiar English characters with unfamiliar Chinese characters while still using singleton targets, to determine whether intertrial repetition of the character identity will still lead to a benefit in RTs if we reduce the likelihood of “compulsive encoding.” If the use of unfamiliar Chinese characters eliminated intertrial target repetition benefits, this would provide converging evidence that detection performance is unaffected by shifts of focal attention.

### Methods

Sixteen Johns Hopkins University undergraduate students (mean age = 19.7 years; 11 male) with normal or corrected-to-normal visual acuity and normal color vision participated in sessions lasting 30–60 min. Participants received extra credit in undergraduate courses as compensation. Participants gave informed consent, and the protocol was approved by the Johns Hopkins Homewood Institutional Review Board.

In Experiment 3A, Chinese characters were used instead of English letters. These characters came from the “Yung” Chinese font (Pelli et al., 2006). In all other respects, Experiment 3A was identical to the singleton-target condition of Experiment 1 (see Figure 1). Participants were given a questionnaire regarding the English translations of the Chinese characters at the conclusion of the experiment in order to assess the participants' Chinese reading comprehension skills. The one participant who was able to identify a subset of the characters was replaced. In all other respects, the design was identical to Experiment 1.

### Results and discussion

We eliminated all responses faster than 100 ms and subsequently used a modified recursive trimming procedure (Van Selst and Jolicoeur, 1994) to remove outliers in each experimental condition. This resulted in an elimination of 1.2% of all trials. All error trials were removed for RT analysis (2.1% of all trials). Because we were comparing intertrial effects resulting from repeated target properties, the following analyses only include trials where a target was present on consecutive trials and the observers' response on the previous trial was correct. Performance accuracy was again high (97.9%).

**Figure 6:**
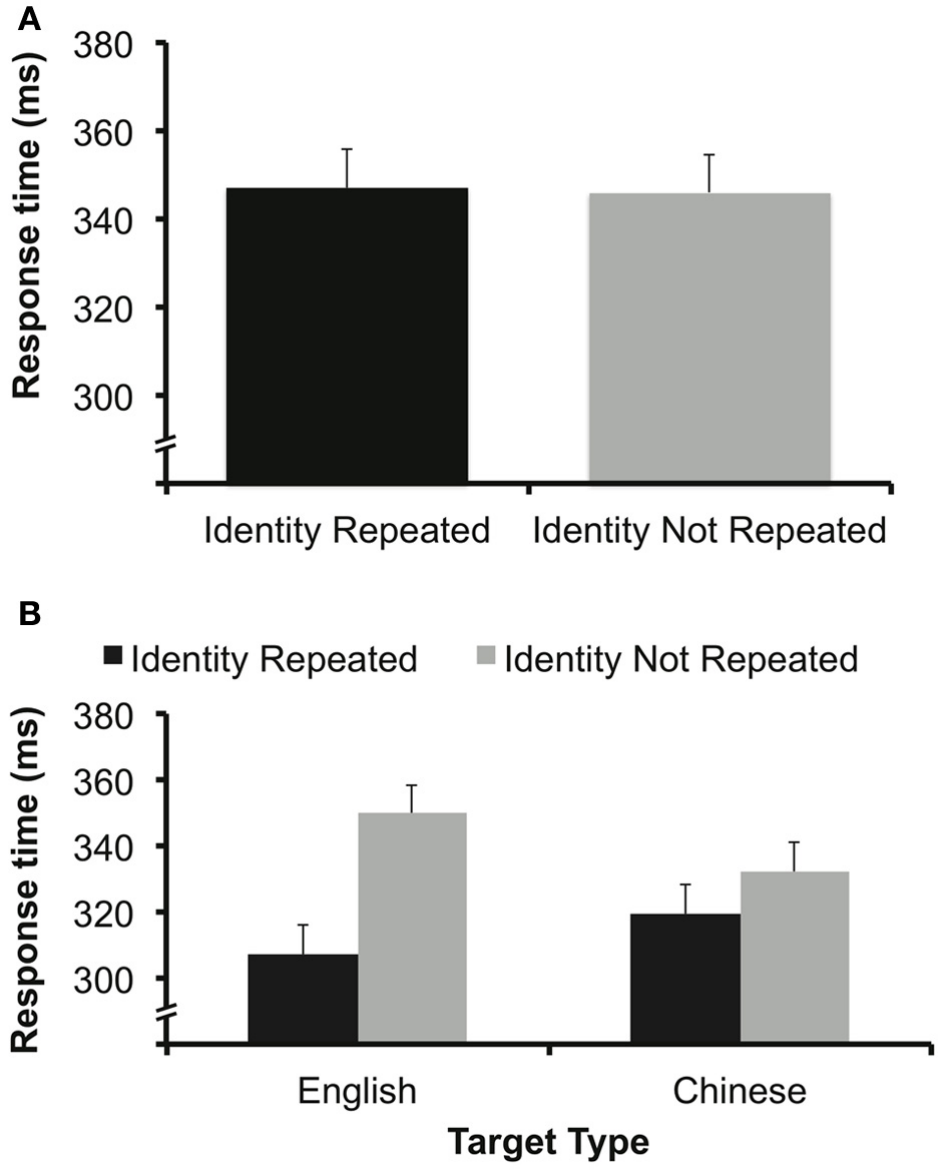
**(A)** Data from Experiment 3A. There was no effect of target identity repetition. Error bars represent 95% confidence interval (e.g., Loftus and Masson, 1994). **(B)** Data from Experiment 3B. There was a benefit to repeating target identity for both English letters and Chinese letters. Error bars calculated from a within-subjects interaction error term (e.g., Loftus and Masson, 1994).

We conducted a one-way ANOVA with a within-subject factor of target identity repetition (identity repeated vs. not repeated) for measures of ER and RT. Again, there was no significant effect of target repetition on RT (347 ms for repetition trials, 346 ms for non-repetition trials), *F*_(1, 15)_ < 1 (Figure 6A), or ER (1.2% for repetition trials, 2.3% for non-repetition trials), *F*_(1, 15)_ = 3.24, *p* > 0.09. As in Experiment 2, this is a case where a singleton target was easily detected but did not result in repetition priming, suggesting a dissociation between the process related to detection and those related to shifts of focal attention that elicit repetition priming effects.

We note the possibility that the target identity repetition benefits observed in the singleton condition of Experiment 1 and Theeuwes et al. (2008) could reflect semantic priming rather than perceptual priming. If that were the case, we would not expect priming to occur in the present study regardless of whether observers shifted focal attention to the target or not, because the characters used in the present study had no semantic association.

A study by Petit et al. (2006) used electroencephalographic measures to determine the time-course of single letter priming effects examining both visual similarity and case-independent letter identity. Petit et al. found that visual similarity to a previous letter affected the electrophysiological response much earlier in time (120–180 ms) than case-independent letter identity effects (220–300 ms). Eddy et al. (2006) found similar evidence for early modulation of electrophysiological responses based on shared perceptual properties using non-letter object stimuli. Thus, it seems likely that target identity repetition effects observed in the present study and Theeuwes et al. (2008) are attributable at least in part to perceptual priming. Still, further studies would be necessary to rule out entirely the possibility that the target identity repetition effects observed in Experiment 1 and Theeuwes et al. (2008) were due to semantic priming alone.

## Experiment 3B

In Experiment 3A, it is possible that focal attention was directed to the letter, but because of the complexity of the Chinese characters we used, participants were unable to fully process the identity of the stimuli. Perhaps the Chinese characters used in this experiment were equivalent for our participants to the “impossible objects,” used by Schacter et al. (1990). In that study, participants had to determine whether presented objects could possibly exist in 3-dimensional space, and there was no benefit for repeating impossible objects on consecutive trials. Had we used impossible objects as the red target, participants might have shifted focal attention to the impossible object target, but we would not observe intertrial facilitation when the same impossible object was repeated. We would therefore not be able to use that lack of intertrial priming to conclude anything about focal attention.

To address this possible confound, we ran an additional experiment in which participants saw a single letter presented at the center of the screen, and were asked to identify whether the letter was an English letter or not. The purpose of this experiment was to determine whether any intertrial facilitation was possible with unfamiliar Chinese characters in our subject population.

### Methods

Sixteen Johns Hopkins University undergraduate students (mean age = 19.8 years; 5 male) with normal or corrected-to-normal visual acuity and normal color vision participated in sessions lasting 30–60 min. Participants received extra credit in undergraduate courses as compensation. Participants gave informed consent, and the protocol was approved by the Johns Hopkins Homewood Institutional Review Board.

Participants indicated whether a centrally presented white character was in English or Chinese with a button press. They pressed the “z” key if it was English and the “x” key if it was not English. Non-English letters were always Chinese characters. The letter subtended 1° of visual angle, and was presented for 120 ms. When the language of the letter was the same on consecutive trials (which was the case 50% of the time), the identity of the letter was repeated 50% of the time. As in Experiment 3A, participants were given a questionnaire regarding the English translations of the Chinese characters at the conclusion of the experiment in order to assess the participants' Chinese reading comprehension skills, and only participants who did not correctly identify any of the Chinese characters were included in the study. There were 10 blocks of 64 trials each in the experiment, the first of which was a practice block.

### Results and discussion

We eliminated all responses faster than 100 ms and subsequently used a modified recursive trimming procedure (Van Selst and Jolicoeur, 1994) to remove outliers in each experimental condition. This resulted in an elimination of 1% of all trials. All error trials were removed for RT analysis (3% of all trials). Because we were comparing intertrial effects resulting from repeated target properties, the following analyses only include trials where the language of the target was repeated on consecutive trials, and the observers' response on the previous trial was correct.

We performed a 2 × 2 ANOVA with within-subjects factors of character language (English or Chinese) and target identity repetition (identity repeated vs. not repeated) for measures of ER and RT. There was no main effect of language on RT, *F*_(1, 15)_ < 1, or ER, *F*_(1, 15)_ = 2.8, *p* > 0.1. There was a main effect of repeating the identity of the character on both RT, *F*_(1, 15)_ = 48.12, *p* < 0.001, and ER, *F*_(1, 15)_ = 22.06, *p* < 0.001. RT was faster (313 vs. 341 ms) and participants made fewer errors (1.6 vs. 3.6%) when the identity of the target was repeated. There was also a significant interaction between character language and target identity repetition for RT, *F*_(1, 15)_ = 12.75, *p* < 0.01 (Figure 6B). There was a greater RT benefit when an English character was repeated (43 ms) than when a Chinese character was repeated (13 ms). There was no interaction for ER, *F*_(1, 15)_ < 1.

We analyzed target identity repetition benefits for the different languages separately with simple main effects analyses. The benefit of repeating the target character on RT was significant for both English characters (307 vs. 350 ms), *F*_(1, 15)_ = 54.39, *p* < 0.001, and Chinese characters (319 vs. 332 ms), *F*_(1, 15)_ = 4.9, *p* < 0.05. The benefit for ER was significant for English characters (2.1 vs. 4.2%), *F*_(1, 15)_ = 10.62, *p* < 0.01 and for Chinese characters (1.1 vs. 3%), *F*_(1, 15)_ = 8.65, *p* < 0.05.

This task differed from the task in Experiment 3A in that observers were indicating the language of the letter, the letter was presented centrally rather than peripherally, and the location of the letter (at fixation) was known in advance of each trial. Therefore, we cannot be certain that these results would generalize to the display characteristics of Experiment 3A. Nevertheless, these results demonstrate that some type of priming is possible with unfamiliar Chinese characters, suggesting that the characters are not so complex as to preclude any form of priming. Therefore, these results suggest that the complexity of the characters *per se* is likely not responsible for the lack of intertrial facilitation observed in Experiment 3A.

## General discussion

Repeating the identity of a color singleton target letter on consecutive trials sped detection RT, replicating the results of Theeuwes et al. (2008). This suggests that focal attention was directed to the target letter, allowing identification of that letter before execution of the detection response. However, this repetition benefit disappeared when the target letter was not a color singleton, while detection performance remained high. The target identity repetition benefit also disappeared in the singleton feature target detection task when participants were required to perform a simultaneous resource-limited central task (Experiment 2) and when the English letters were replaced with unfamiliar Chinese characters (Experiment 3).

Target identity repetition effects changed across the different conditions in three experiments, even though the target-defining feature was identical in all conditions. Thus, disparities emerged only because of changes in how focal attention was (or was not) deployed to the target letter, and the deployment of focal attention was determined by factors such as the perceptual salience of the target (Experiment 1), the availability of excess cognitive resources (Experiment 2), or the familiarity of the stimuli (Experiment 3). Together, these data demonstrate that detection performance was unaffected by changes in the deployment of focal attention. This suggests that detection occurred without the deployment of focal attention in the non-singleton condition of Experiment 1, and Experiments 2 and 3. However, proving a negative is difficult. Although we demonstrate converging evidence, we cannot with absolute certainly claim that detection occurs in the complete absence of focal attention based on these null results. Instead, in the following section, we consider how shifts of focal attention might differ depending on context, and how those differences relate to the detection process. This discussion focuses primarily on the salience manipulation from Experiment 1.

### The relationship between focal attention and detection

A singleton target is likely to have a stronger pull on attention than a non-singleton target because a singleton target conveys a more robust bottom–up signal to the observer's visual system (cf. Bravo and Nakayama, 1992). This might lead to differences in the selection process for each target by affecting the *speed* of the deployment of focal attention. In that case, the signal from a singleton target would pass a decision threshold more quickly than the signal from a non-singleton target, and a singleton target would be selected at an earlier point in time relative to its onset compared to a non-singleton target.

Additionally, the *strength* of the selection process following deployment of focal attention could be altered by target salience. That is, focal attention could be deployed to a target at roughly the same delay regardless of the target's bottom–up salience, but more attentional resources might be deployed when the target is a singleton. Subsequently, more information would be extracted from the target at a quicker pace immediately following selection when the target is a singleton rather than a non-singleton.

We can consider how these processes relate to the detection process in Experiment 1. One explanation for our results is that a shift of focal attention preceded detection whether the target was a color singleton or not, but that the selection process itself was robust enough to result in letter identification prior to detection only in the singleton-target condition of Experiment 1. This account would not be tenable if only the *speed* of deployment of focal attention differed between the two conditions, because if the selection process itself were the same between the two conditions once it reached the target, letter identification should precede color detection in both cases and overall RT would be slower in the non-singleton-target condition. However, neither of these results occurred. If the *strength* of selection differed between the two conditions, we would also have to conclude that changing the *strength* of the selection process influenced letter identification but not detection. Thus, detection and identification would constitute independent components of the selection process that are differentially affected when the strength of deployment changes.

A more parsimonious explanation of our results, which also fits well with the data from Experiments 2 and 3, is simply that detection occurs independently from focal attention, as proposed in FIT (Treisman and Gelade, 1980). When a target is defined by a single feature, deployment of attention to that target may depend on factors such as perceptual salience, availability of excess attentional resources, or the familiarity of the target. In some cases, selection occurs quickly and robustly enough to precede the detection process (as in the singleton-target condition, and Theeuwes et al., 2008), but this does not mean that selection always necessarily precedes detection. It is not surprising that focal attention would eventually be directed toward the target in the non-singleton-target condition in Experiment 1, for example, because excess attentional resources were likely available due to the simple nature of the task. Thus, we would infer that focal attention was likely deployed toward the target in the non-singleton-target condition, but that the detection process was completed independently and concluded before focal attention reached the target.

The latter interpretation is consistent with data from a recent study by Turatto et al. (2010). In that study, observers were presented with a ring of colored circles and cued to search for a particular target color on each trial. In the *detection task*, observers had to determine whether any of the circles matched the target color. In the *discrimination task*, observers had to indicate whether the letter inside one of the circles was a consonant or vowel. For both task types, there were three possible types of target-present displays: a color singleton target display (i.e., one of the circles matched the target color, and the remaining circles matched the distractor color), a color singleton distractor display (i.e., one of the circles matched the distractor color, and the remaining circles matched the target color), or a no-singleton display (all circles were target-colored).

Response times in the detection task were slowest when there was only one target (the color singleton target). On the other hand, RTs in the discrimination task were faster, and search slopes were shallower, on color singleton target displays compared to color singleton distractor displays, even though more target circles were present in the singleton distractor displays. Because the discrimination task requires processing of a letter stimulus at a specific location, a shift of focal attention is required. As expected, the data demonstrate that bottom–up salience influences this process, as RTs were faster when the target stimulus was salient in the discrimination task. However, in the detection task, salience was not a factor; instead, the number of target items determined how fast observers detected the presence of a target-colored circle. Thus, the data suggest that detection is not influenced by target selection, and instead that detection may occur without a shift of focal attention.

### Letter identification without a shift of focal attention?

In the Singleton Condition of Experiment 1, we presumed that because we found intertrial identity repetition benefits, the target letter was selected following a shift of focal attention. However, the data from the present studies might be interpreted in the framework of other attention theories, such as load theory (e.g., Lavie, 1995) or dilution (e.g., Tsal and Benoni, 2010) that do not necessarily require a shift of focal attention for letter identification. Unfortunately, load and dilution studies have typically used tasks that require explicit identification of letters, rather than a color target detection task that we use here in which letter processing is likely implicit and is not task-relevant. Therefore, it is difficult to interpret exactly how load and dilution theories would be used to predict the current results. For example, it may be the case that the singleton-target condition of Experiment 1 reflected a reduced perceptual load or reduced dilution from non-target distractors relative to the non-singleton-target condition, thus explaining why the target letter's identity was processed before the detection response. On the other hand, the number of neutral distractor letters is identical in both conditions of Experiment 1, and previous studies supporting the dilution account (e.g., Benoni and Tsal, 2010; Tsal and Benoni, 2010) have suggested that the number of neutral distractor letters present is a determining factor in whether or not processing of the target's identity is affected by distractor presence.

Additional studies would be useful in addressing how the results of the current study might be informed by load and dilution theories, and might potentially challenge the claim that intertrial priming in the current paradigm reflects a shift of focal attention. However, even if the target repetition effects in the present study did not reflect a shift of focal attention, the results reported here would still demonstrate that changes in target salience, availability of excess cognitive resources, or target familiarity affect intertrial repetition priming effects without affecting detection performance. This demonstrates that the processes and resources involved in letter identification are not required for the detection of a simple feature target.

## Conclusion

It has been argued that even a simple feature target can only be detected after that target has been selected by focal attention. Here we present new evidence that brings that claim into question. Specifically, in three experiments, we have shown that whether or not letter identity is processed before the detection response, a direct result of selection by focal attention, does not affect detection performance; therefore, there is a minimal or absent relationship between focal attention and simple feature detection. Instead, whether simple feature targets are selected prior to detection depends on factors such as the perceptual salience of the target (Experiment 1), the availability of excess cognitive resources (Experiment 2), and the familiarity of the target stimulus set (Experiment 3).

### Conflict of interest statement

The authors declare that the research was conducted in the absence of any commercial or financial relationships that could be construed as a potential conflict of interest.

## Footnotes

^1^In a separate study, we varied the number of items on a trial-by-trial basis (2, 4, or 8). Fourteen participants searched for a red target among heterogeneously colored displays similar to those described in the methods section of Experiment 1. We found minimal costs in RT on target-present trials with increasing set size (369, 370, and 380 ms, respectively; slope = 1.9 ms/item). The effect of display size was not significantly different from 0, *F*_(2, 26)_ = 1.68, *p* > 0.1. This result is in line with several previous studies showing minimal search slopes for detection of a simple color target in a heterogeneous display (e.g., Duncan, 1989; D'Zmura, 1991; Bauer et al., 1996).
